# Paeonol alleviates neuropathic pain by modulating microglial M1 and M2 polarization via the RhoA/p38MAPK signaling pathway

**DOI:** 10.1111/cns.14211

**Published:** 2023-04-10

**Authors:** Xin Li, Huimei Shi, Di Zhang, Bei Jing, Zhenni Chen, Yachun Zheng, Shiquan Chang, Li Gao, Guoping Zhao

**Affiliations:** ^1^ College of Traditional Chinese Medicine Jinan University Guangzhou China

**Keywords:** CCI, p38MAPK, Paeonol, Pseudotime, RhoA, single‐cell sequencing

## Abstract

**Background:**

This study aimed to investigate the potential mechanism of paeonol in the treatment of neuropathic pain.

**Methods:**

Relevant mechanisms were explored through microglial pseudotime analysis and the use of specific inhibitors in cell experiments. In animal experiments, 32 SD rats were randomly divided into the sham operation group, the chronic constrictive injury (CCI) group, the ibuprofen group, and the paeonol group. We performed behavioral testing, ELISA, PCR, Western blotting, immunohistochemistry, and immunofluorescence analysis.

**Results:**

The pseudotime analysis of microglia found that RhoA, Rock1, and p38MAPK were highly expressed in activated microglia, and the expression patterns of these genes were consistent with the expression trends of the M1 markers CD32 and CD86. Paeonol decreased the levels of M1 markers (IL1β, iNOS, CD32, IL6) and increased the levels of M2 markers (IL10, CD206, ARG‐1) in LPS‐induced microglia. The expression of iNOS, IL1β, RhoA, and Rock1 was significantly increased in LPS‐treated microglia, while paeonol decreased the expression of these proteins. Thermal hyperalgesia occurred after CCI surgery, and paeonol provided relief. In addition, paeonol decreased the levels of IL1β and IL8 and increased the levels of IL4 and TGF‐β in the serum of CCI rats. Paeonol decreased expression levels of M1 markers and increased expression levels of M2 markers in the spinal cord. Paeonol decreased IBA‐1, IL1β, RhoA, RhoA‐GTP, COX2, Rock1, and p‐p38MAPK levels in the spinal dorsal horn.

**Conclusion:**

Paeonol relieves neuropathic pain by modulating microglial M1 and M2 phenotypes through the RhoA/p38 MAPK pathway.

## Graphical abstract

Experiment flow chart.

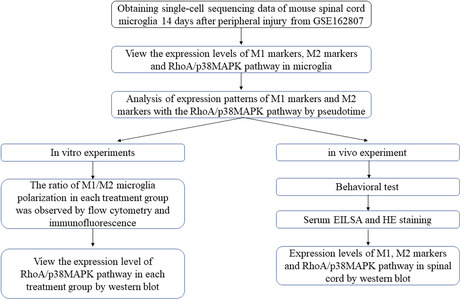

## 1 INTRODUCTION

Neuropathic pain (NP) is pain caused by injury or disease of the somatosensory nervous system.^1^ A total of 6.9%–10% of the world's population suffers from NP.^2^ Patients experience NP even after the initial stimulus has been removed.^3^ The National Institute for Health and Clinical Excellence (NICE) guidelines recommend NSAIDs for neuropathic pain relief,^4^ but NSAID resistance and long‐term side effects continue to plague patients.^5^, ^6^, ^7^ Therefore, there is an urgent need for novel medicine for the treatment of NP.

Microglia are resident macrophage‐like immune cells in the central nervous system thought to play an important role in NP.^8^ Numerous studies have shown that inhibition of microglial activation can alleviate NP.^9^, ^10^, ^11^, ^12^ Microglia can be activated into the M1 phenotype and the M2 phenotype,^13^ in which the M1 phenotype is mainly involved in pro‐inflammatory responses and the M2 phenotype is mainly involved in anti‐inflammatory responses.^14^ Inflammatory factors such as IL1β and TNF‐α secreted by M1 phenotype microglia mediate the abnormal excitability of spinal neurons leading to hyperalgesia,^8^ while M2 phenotype microglia produce opioid peptides to reduce pain.^15^ Therefore, converting the M1 phenotype to the M2 phenotype may be a strategy for treating NP.

RhoA and Rho‐related kinase (Rho kinase; ROCK) play key roles in neuropathic pain.^16^, ^17^ Studies have shown that ROCK inhibition reduces thermal hyperalgesia induced by TRP channels.^18^ The ROCK pathway may lower the spinal cord nociceptor threshold and be involved in the development and maintenance of neuropathic pain.^19^ RhoA/p38MAPK activates microglia in the spinal cord and increases neural excitability through P2Y12 receptors.^20^

Paeonol is an anti‐inflammatory and antioxidant compound widely distributed in Paeonia lactiflora. Several studies have shown that paeonol can relieve pain in mouse and rat models.^21^, ^22^ Paeonol can inhibit the expression of inflammatory factors in microglia, which indicates that paeonol can relieve NP through microglia. However, whether paeonol can regulate microglial M1 and M2 polarization and the underlying mechanism in NP remains unknown.

The purpose of this study was to determine the therapeutic effect of Paeonol on neuralgia and related mechanisms. We investigated the underlying mechanisms at the cellular level through single‐cell Pseudotime analysis and in vitro experiments. The therapeutic effect of paeonol on the CCI model was evaluated by behavioral examination, pathological examination, and immunohistochemistry.

## 2 MATERIALS AND METHODS

### 2.1 Reagents

Paeonol (H111081; 99% purity) was acquired from Shanghai Aladdin Biotechnology Co., Ltd. (China). Ibuprofen (lot number: H20066) was purchased from Guangzhou Overseas Hospital, First Affiliated Hospital of Jinan University, Ltd. LPS (L2880) and CCG‐1423 (an inhibitor of RhoA, T2014) were acquired from Guangzhou Yiyou Biotechnology Biological Co., Ltd. SB203580 (an inhibitor of p38 MAPK) was purchased from Selleck Chemicals (Shanghai, China). SYBR Green Premix qPCR Kit and Evo M‐MLV RT Mix Kit (AG11701 and AG11012, respectively) were purchased from Accurate Biotechnology Co., Ltd. A Cell Membrane Protein and Cytoplasmic Protein Extraction Kit (BL671A) was purchased from Biosharp Biotechnology Co., Ltd. (China). RIPA buffer (WB‐0071) was purchased from Beijing Dinguo Biological Co., Ltd., and polyvinylidene fluoride membranes were purchased from Millipore (Billerica, USA). IL1β (MM‐0047R1), IL4 (MM‐0191R2), IL8 (MM‐20594R1), INFγ (MM‐0198R1), and TGFβ (MM‐20594R1) ELISA kits were purchased from American Cotton Industry Co., Ltd. (China). The antibodies used were as follows: Rhoa (A5651), p38MAPK (86900S), p‐p38MAPK (4511S), ROCK1 (A5141), ROCK2 (A5156), IL1β (511369), iNOS (2982S), Arg‐1 (93668), COX2 (K0619), IBA‐1 (A5141) β‐actin (4970S), and goat anti‐rabbit IgG (H + L) HRP (E030120‐02). Anti‐rat CD206 APC (bs‐4727R) was obtained from Bioss (Beijing, China). PE anti‐mouse CD32 (Fcgr2) (156404) and APC anti‐mouse CD206 (MMR) (141708) were purchased from Biolegend (Beijing) Biotechnology Co., Ltd.

### 2.2 Subjects

Thirty‐two male Sprague–Dawley rats aged 7–8 weeks and weighing approximately 180–230 g were obtained from the experimental center of Beijing Huafukang Co., Ltd. These rats were housed 5 per cage at the Animal Experimental Center of the Medical College of Jinan University. All animal experiments were conducted in accordance with the guidelines established by the National Academy of Sciences and the National Institutes of Health (NIH) (Institute of Laboratory Animal Resources (U.S.)), the Committee on Care and Use of Laboratory Animals, and the Animal Ethical Committee of Jinan University (ethics approval number: IACUC‐20220314‐12).

### 2.3 NP model

Based on previous studies, we chose to generate the chronic contractile injury (CCI) pattern of the sciatic nerve as a disease model for sciatica. The right sciatic nerve of rats was exposed following an intraperitoneal injection of sodium pentobarbital (3%; 40 mg/kg), and the right sciatic nerve was ligated under a microscope using 4.0 sutures (repeated 4 times at ~1 mm intervals). Rats in the sham‐operated group were not subjected to nerve ligation. To prevent infection, gentamicin (10 mg/mL) was injected into the right biceps femoris muscle. A total of 18 rats were randomized into two groups: the CCI and sham operation groups. After 21 days, the rats were sacrificed with an overdose of sodium pentobarbital (200 mg/kg).

### 2.4 Single‐cell RNA‐seq data processing and quality control

Single‐cell sequencing data of mouse spinal cord microglia 14 days after peripheral nerve injury were obtained from the sequencing data of Mouse microglia 207‐R, Mouse microglia 210, and Mouse microglia 212 in the GEO database GSE162807 cohort. It contains transcriptome sequencing data for 9055 cells. To ensure the reliability of the data analysis, we removed low‐quality cells and retained cells with more than 200 genes but fewer than 4000 genes and fewer than 20% mitochondrial genes. We then applied the Seurat R package for quality control. Gene expression measurements per cell were normalized by total expression using the global scaling normalization method “LogNormalize,” and then the results were multiplied by a scaling factor (default 10,000) and log‐transformed in each dataset. We annotated the data using the built‐in dataset “ImmGenData” in the “SingleR” package, which resulted in the identification of 8633 microglia, 8 endothelial cells, 1 fibroblast, 6 macrophages, 9 monocytes, and 2 neutrophils. We selected microglia for further analysis. Afterward, we performed principal component analysis (PCA) on highly variable genes and performed dimensionality reduction on single‐cell data by the Uniform Manifold Approximation and Projection (UAMP) method “Monocle2” for differentiation trajectories and pseudotemporal analysis of microglia.

### 2.5 Molecular docking

3D protein structures were downloaded from the RCSB PDB database (http://rcsb.org/). The molecular structure of the ligand was downloaded from the Traditional Chinese Medicine System Pharmacology Database and Analysis Platform (TCMSP). We used PyMol software to remove all water molecules from the 3D structure of the protein and added polar hydrogens and Gasteiger charges to the protein and ligands using AutoDockTools 1.5.6. The AutoDock tool is used to match compounds and genes, where AutoDock Vina is used to find optimal docking conditions. Docking energies were used to evaluate the results of molecular docking. PyMol enables analysis and visualization of docking results.

### 2.6 Cell viability assay

GMI‐R1 cells (rat microglia) were purchased from Huatuo Biotechnology. The cell lines used in the experiments were between passages 4 and 10. The CCK‐8 assay was performed to assess cell viability after paeonol treatment. According to the instructions of the CCK8 reagent, we tested the effect of paeonol at concentrations of 1, 2, 4, 8, 10, 15, and 20 μM on the viability of GMI‐R1 cells.

### 2.7 Cell grouping and drug concentration

The cells were divided into 6 groups: the control group, the LPS group (10 μg/mL LPS), the CCG group (10 μg/mL LPS + 10 μM CCG‐1423), the SB203580 group (10 μg/mL LPS + 10 μM SB203580),^18^ the LPS + paeonol group (10 μg/mL LPS + 4 μM paeonol), and the paeonol group (4 μM paeonol). GMI‐R1 cells were cultured in 6‐well plates for 24 h. The medium was then discarded, and the cells were washed with PBS. The corresponding drugs and LPS were added to the medium at the same time, and then the cells were cultured for 24 h.

### 2.8 Flow cytometry analysis

Cells were seeded in 6‐well dishes at a density of 1 × 10^6^ per well and treated with drugs and LPS for 24 h. Cells were then digested in the dishes by trypsinization, washed, and resuspended in cold PBS. The membrane protein CD32 was detected by direct immunofluorescence staining, and the cells were incubated with PE‐conjugated CD32 antibody for 30 min at room temperature in the dark. For CD206, cells were fixed and then permeabilized with intracellular staining and washing buffer, and then cells were incubated with APC‐conjugated CD206 antibody for 30 min at room temperature in the dark. Cells were washed twice with PBS and then resuspended in 500 μL of PBS. The light‐scattering properties of each sample (10^5^ cells) were analyzed using a flow cytometer (Cyto‐FLEX, Beckman Coulter, USA) equipped with FlowJo software.

### 2.9 Immunofluorescence

Glass slides coated with poly‐L‐lysine (0.1 mg/mL) were placed in a 24‐well plate. GMI‐R1 cells were cultured and processed as instructed, fixed with 4% paraformaldehyde for 30 min at room temperature, and then permeabilized with 0.3% Triton X‐100 for 15 min. Cells were blocked with PBS containing 10% donkey serum for 1 h. Cells were then incubated with rabbit anti‐CD32 and CD206 primary antibodies (1:200) in a humidified chamber at 4°C overnight. The next day, the cells were washed with PBS and incubated with goat anti‐rabbit secondary antibody (1:400) for 50 min at room temperature. After that, the slides were washed with PBS, and DAPI staining solution was added. After incubation in the dark at room temperature for 10 min, the slides were mounted with anti‐fluorescence quenching mounting medium. All images were captured with a fluorescence microscope (OLYMPUS, BX53).

### 2.10 Animal treatment

Thirty‐two rats were randomly divided into the sham operation group, the CCI group, the ibuprofen group and the paeonol group, with 8 rats in each group. Rats in the sham operation group and CCI group were given normal saline (0.9%, 6 mL/kg/bid), the ibuprofen group was given ibuprofen (31.5 mg/kg/bid) and the paeonol group was given paeonol (100 mg/kg/bid) by intragastric administration,^23^ starting on the first day after surgery and continuing for 21 days.

### 2.11 Hot plate experiment

Thermal hyperalgesia was evaluated by the hot plate test (YLS 6B; Jinan Yiyan Technology Development Co., Ltd.). To measure the paw withdrawal threshold (PWT), the lateral plantar surface of the right paw was placed on a heating plate (50°C), and the time required for the rat to lift the right foot was recorded. PTW values were recorded on the first day before surgery and on days 1, 4, 7, 14, and 21 after surgery.

### 2.12 H&E staining

We fixed ligated sciatic nerve tissues in 4% paraformaldehyde for 1 day, embedded them in paraffin, and sectioned them at 3 μm thickness. Sections were deparaffinized in xylene and rehydrated by 100, 90, 80, and 70% ethanol. We then rinsed the slices in PBS for 5 min. After staining, two pathologists blinded to the experimental design viewed the stained images and assessed tissue damage.

### 2.13 ELISA

Rat serum frozen in a −80°C refrigerator was taken out to detect the changes of IL1β, IL8, INF‐γ, IL4, and TGF‐β inflammatory factors. With the help of ELISA kit, the experimental operation was carried out according to the manufacturer's instructions. Set up blank and standard wells on the enzyme‐labeled plate, add 50 μL of standard products of different concentrations to each of the standard wells, add 50 μL of serum sample diluent (diluted 5 times) to the sample wells to be tested, and add as much as possible to the enzyme label when adding samples. At the bottom of the plate, add 100 μL of enzyme‐labeled reagent to each well, shake gently to mix, seal the plate, and incubate on a shaker at 37°C for 1 h. After removing the liquid, wash with PBS three times, shake dry, add 50 μL of chromogen to each well, incubate at 37°C in the dark for 20 min, and add 50 μL of stop solution (blue turns yellow). Use a microplate reader to detect the absorbance within 15 min, draw a standard curve according to the absorbance of the standard substance, calculate the content of serum inflammatory factors after dilution, and then calculate the level of inflammatory factors when undiluted.

### 2.14 Immunohistochemistry

The right sciatic nerve was fixed in 4% paraformaldehyde for 24 h, immersed in 20% (w/v) sucrose solution, sectioned, and then slices from individual animals were incubated in sodium citrate antigen retrieval solution (1:1000 dilution; pH = 6). Next, sections were incubated with primary and secondary antibodies. Then, DAB was added. Finally, the slides were washed in distilled water, dehydrated, made transparent, and sealed with xylene. The slices were examined under an OLYMPUS fluorescence microscope (BX53). The mean density was calculated using Image‐Pro Plus software (Media Cybernetics, Inc.) and statistical analysis was performed.

### 2.15 Quantitative real‐time PCR (qRT–PCR)

RNA was extracted using RNAiso Plus and reverse‐transcribed to cDNA using an RT–qPCR kit according to the manufacturer's instructions. The amplification parameters were 95°C for 1 min, followed by 40 cycles of 95°C for 10 sec and 60°C for 30 s. The relative gene expression data were analyzed by RT–qPCR using the 2^−ΔΔCT^ method. The primers used are listed in Table 1.

**TABLE 1:** Primer sequences.

Gene	Forward primer (5′ → 3′)	Reverse primer (5′ → 3′)
Β‐actin	CCTAGACTTCGAGCAAGAGA	GGAAGGAAGGCTGGAAGA
TNF‐α	GCGTGTTCATCCGTTCTCTACC	TACTTCAGCGTCTCGTGTGTTTCT
Arg‐1	CAGTATTCACCCCGGCTA	CCTCTGGTGTCTTCCCAA
iNOS	CGGAGAACAGCAGAGTTGG	GGAATAGCACCTGGGGTTT
IL6	AGTTGCCTTCTTGGGACTGATGT	GGTCTGTTGTGGGTGGTATCCTC
IL1β	AGGAGAGACAAGCAACGACA	CTTTTCCATCTTCTTCTTTGGGTAT
CD32	CCCACAACACCAAGAACTG	CTGATGCCGGTCTCCTC

### 2.16 Western blotting

The right spinal dorsal horn of the rat was homogenized in RIPA buffer containing 1 mM of phenylmethylsulfonyl fluoride (PMSF) and centrifuged at 14,000× for 20 min. Total protein was extracted from GMI‐R1 cells in RIPA lysis buffer containing 1 mM of PMSF. We acquired the membrane fraction using a cell membrane protein and cytoplasmic protein extraction kit. The proteins (30 μg) were separated by 12% SDS‐PAGE and transferred onto PVDF membranes; the membranes were blocked with 5% skimmed milk powder for 1 h, incubated with primary antibody (1:1000) overnight at 4°C for 24 h, and incubated with secondary antibody (1:30,000) for 1 h. A ChemiDoc XRS imager was then used to visualize the bands. Each experiment was conducted in triplicate.

### 2.17 Statistics

Data are reported as mean ± standard error (SEM) of three independent experiments, each performed in triplicate, and analyzed using GraphPad Prism 9 and R 4.0.1 software. Normality tests were performed using Kolmogorov–Smirnov. If the data are normally distributed with uniform variance, perform one‐way analysis of variance (ANOVA) and two‐way ANOVA followed by post hoc (Bonferroni) tests for multiple group comparisons. Nonparametric tests were used if the data did not conform to a normal distribution. *p*‐value < 0.05 was considered statistically significant.

## 3 RESULTS

### 3.1 Expression of the RhoA/p38MAPK pathway in microglia after peripheral nerve injury

**FIGURE 1:**
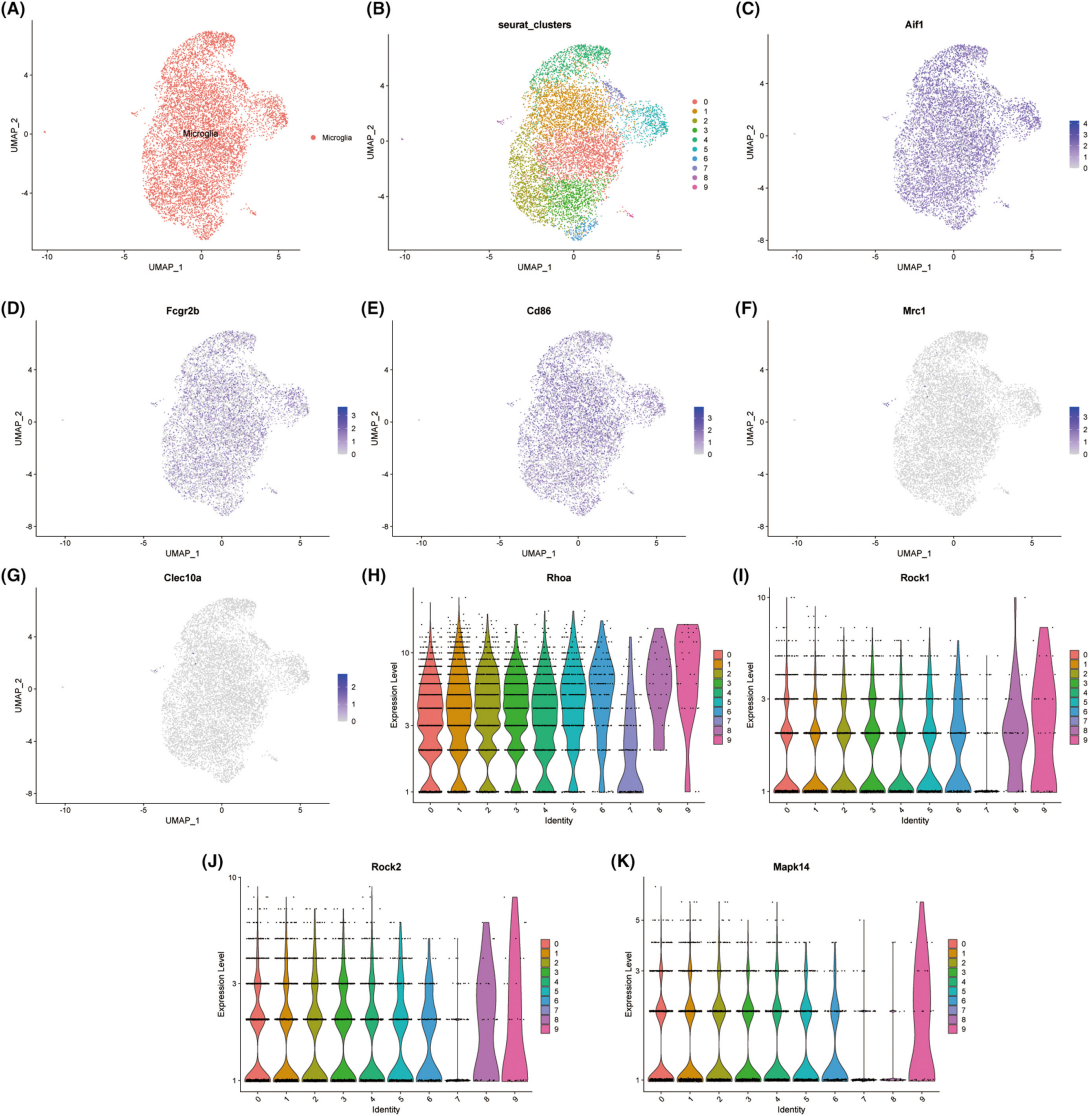
Expression levels of microglia M1, M2 markers and RhoA/p38MAPK expression levels in spinal cord after peripheral nerve injury. (A) Cell types from single‐cell sequencing data. Red dots represent microglia. (B) UAMP plot showing microglia grouped into 9 clusters. (C) UAMP plot of expression levels of microglial activation marker Aif1 (IBA‐1). (D, E) UAMP plot of expression levels of M1 markers Fcgr2b and Cd86 in microglial cells. (F, G) UAMP plot of the expression levels of M2 markers Mrc1 and Clec10a in microglial cells. Purple represents high expression, white represents low expression. (H–K) Violin plot of expression of Rhoa, Rock1, Rock2, and p38MAPK (MAPK14) in microglia.

To gain insight into the role of M1/M2 polarization of macrophages (microglia) in the central nervous system in peripheral nerve injury, we selected the single‐cell sequencing data of spinal cord microglia 14 days after peripheral nerve injury for analysis, because the time point of 14 days is in the late stage of nerve injury, and neuroinflammation and nerve repair exist at the same time. We then performed principal component analysis (PCA) on the microglia in the samples using the “Seurat” R package and clustered them using the nonlinear dimensionality reduction algorithm UAMP. The results showed that microglia could be divided into 9 clusters (Figure 1A,B). We then examined the expression of the microglial activation marker Aif1 (IBA‐1) in each microglial cluster by UAMP plots, and the results showed that Aif1 was highly expressed in microglia in all 9 clusters (Figure 1C), then we further looked at the expression levels of M1 markers Fcgr2b (CD32), Cd86 and M2 markers Mrc1 (CD206), Clec10a in these 9 clusters, and the results showed that M1 markers Fcgr2b and Cd86 are highly expressed in these microglial cells (Figure 1D,E), while the M2 markers Mrc1 and Clec10a were only expressed in a small number of microglial cells (Figure 1F,G), indicating that most of the microglial cells in the spinal cord after peripheral nerve injury were of the M1 phenotype. Next, to further understand the expression of the RhoA/p38MAPK pathway in these M1 phenotype microglia, we examined the expression of Rhoa, Rock1, Rock2, and Mapk14 (p38MAPK) in each microglial cluster by violin plot. We found high expression levels of both Rhoa and Rock1 in microglia in all 9 clusters (Figure 1H,I). Rock2 was highly expressed in the other 8 clusters except the 7th cluster, which had a low expression level (Figure 1J). Mapk14 was highly expressed in the other 7 clusters except the 7th and 8th clusters (Figure 1K).

### 3.2 Differentiation trajectories of microglia after peripheral nerve injury

**FIGURE 2:**
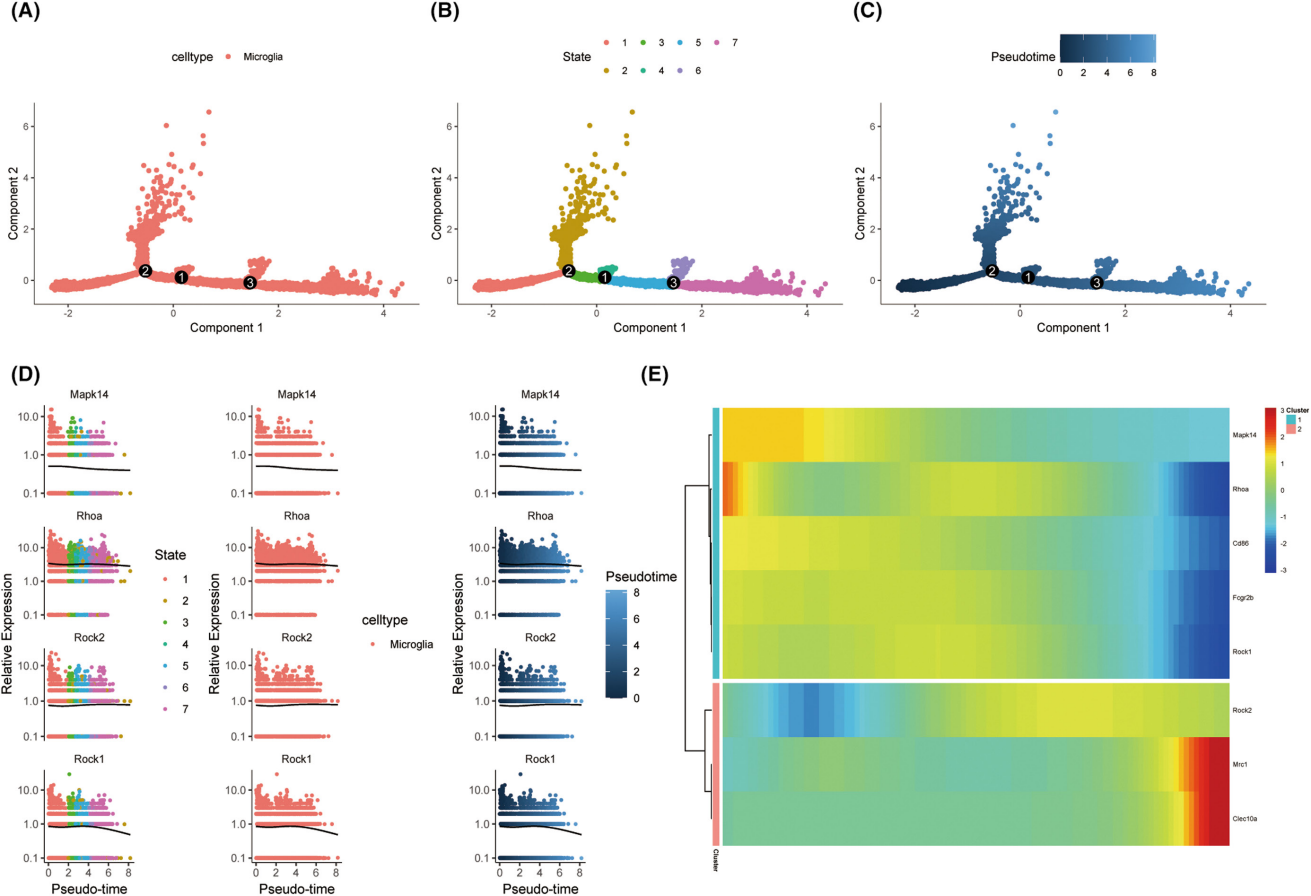
Pseudotime analysis of microglia. (A) Differentiation trajectories of microglia. Display by cell type. (B) Differentiation trajectories of microglia. The abscissa is the pseudotime series. From left to right are microglia from the initial state to the final state. There are 3 branch nodes, divided into 7 states. (C) Microglia initial state to final state is shown. The brighter the blue, the “older” the cell state is in pseudotime. (D) Changes in the expression levels of RhoA, Rock1, Rock2, and MAPK14 in the pseudotime differentiation trajectories of microglia. (E) Clustering and expression trends of RhoA, Rock1, Rock2, and MAPK14 with the M1 markers Fcr2b and CD86 and the M2 markers Mrc1 and Clec10a.

Since the RhoA/p38MAPK pathway is highly expressed in microglia, to further study the biological function of this pathway in the differentiation of microglia, we used “Monocle2” to analyze the differentiation trajectory. We found that microglia could be classified into seven states as the pseudotime course progressed (Figure 2A–C). We examined changes in the RhoA/p38MAPK pathway in microglial differentiation trajectories. We found that Rhoa, Rock1, and Mapk14 were highly expressed in state 1, then the expression levels of Rhoa and Rock1 began to decrease in states 2, 3, and 4, increased in states 5 and 6, and finally in state 7. Mapk14 with Pseudotime changes slowly decrease. Rock 2 shows a slow upward trend from states 1 to 7 (Figure 2D). Then, in order to understand whether the expression trends of proteins in the RhoA/p38 pathway are consistent with the expression trends of M1 and M2 markers. We performed a “DifferentialGeneTest” on the expression pattern of the RhoA/p38 pathway using markers for M1 and M2. We found that the expression pattern of Rhoa, Rock1, and Mapk14 was consistent with that of the M1 markers Fcgr2b (CD32) and CD86, whereas that of Rock2 was consistent with the M2 markers Mrc1 and Clec10a (Figure 2E). This result suggests that the RhoA/p38MAPK pathway may be involved in the activation of microglia with the M1 phenotype.

### 3.3 Validation of the interaction between paeonol and RhoA by molecular docking

**FIGURE 3:**
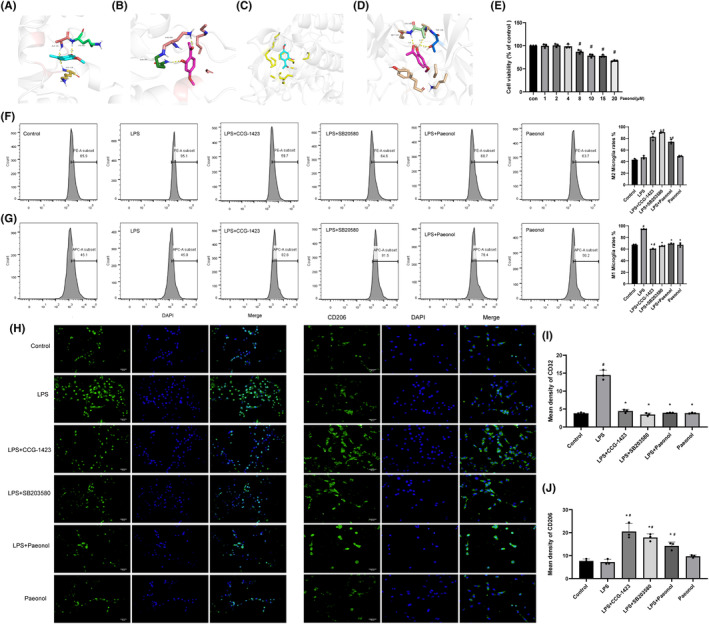
Flow cytometry and immunohistochemistry for M1 and M2 markers. (A–D) Three‐dimensional map of RhoA, Rock1, Rock2, and p38MAPK interactions with paeonol. (E) Determination of the effect of paeonol on the viability of GMI‐R1 cells by the CCK‐8 method. (F) Determination of the proportion of M1 microglia (CD32) by flow cytometry. (G) Determination of the proportion of M2 microglia (CD206) by flow cytometry. (H–J) Immunofluorescence results of CD32 and CD206. #Compared with the control group. *Compared with the LPS group. *n* = 3.

To verify the binding ability of paeonol to the RhoA/p38 MAPK pathway, we performed molecular docking analysis and evaluated the binding energy of the four proteins for paeonol. The binding energy is less than −1.2 kcal/mol, indicating good binding activity. The results showed that paeonol and RhoA proteins interacted stably through three hydrogen bonds (Figure 3A). The results of this analysis are shown in Table 2. The 3D map of the interaction of paeonol with Rock1, Rock2, and p38 MAPK is shown in Figure 3B–D.

**TABLE 2:** Molecular docking of α‐asarone‐TRPs and α‐asarone‐Caspase3.

	Binding energy (kcal/mol)	Binding site	Hydrogen bond length
Paeonol‐RhoA	−5.7	ALA‐161, LYS‐162, LYS‐118	1.9, 1.8, 1.9
Paeonol‐Rock1	−5.4	ASN‐390, GLN‐391	2.0, 2.1
Paeonol‐Rock2	−5.8	None	None
Paeonol‐p38MAPK	−6.0	MET‐109, LEU‐108, THR‐106	1.8, 2.2, 2.7

### 3.4 RhoA, p38MAPK inhibitor, and paeonol convert M1 microglia to M2 microglia in vitro

According to the results of the CCK8 assay, 4 μM of paeonol did not affect the viability of GMI‐R1 cells, but above 8 μM, the cell viability began to decline, so we chose 4 μM for cell experiments. To observe the effect of paeonol on LPS‐induced microglial polarization, the expression of the M1 marker CD32 and the M2 marker CD206 was measured by flow cytometry. The results showed that LPS treatment increased CD32‐positive M1 phenotype microglia compared to the control group, and CCG‐1423 (RhoA inhibitor), SB203580 (p38MAPK inhibitor), and Paeonol reduced LPS‐induced M1 polarization of microglia compared with the LPS group (Figure 3F, *p* < 0.05). CCG‐1423, SB203580, and paeonol increased CD206‐positive M2 phenotype microglia compared to the LPS group (Figure 3G, *p* < 0.05). Moreover, we performed immunofluorescence staining for CD32 and CD206 markers, and the results showed that CCG‐1423, SB203580, and paeonol decreased the expression of CD32 and increased the expression of CD206 in LPS‐treated GMI‐RI1 cells compared with the LPS group (Figure 3H–J, *p* < 0.05). These results suggest that inhibition of RhoA and p38MAPK converts LPS‐treated GMI‐R1 microglia from the M1 phenotype to the M2 phenotype. Paeonol also promoted the transformation of LPS‐treated GMI‐R1 microglia from the M1 phenotype to the M2 phenotype.

### 3.5 Effects of RhoA, p38MAPK inhibitor, and paeonol on inflammatory cytokines produced by LPS‐induced GMI‐R1 cells

**FIGURE 4:**
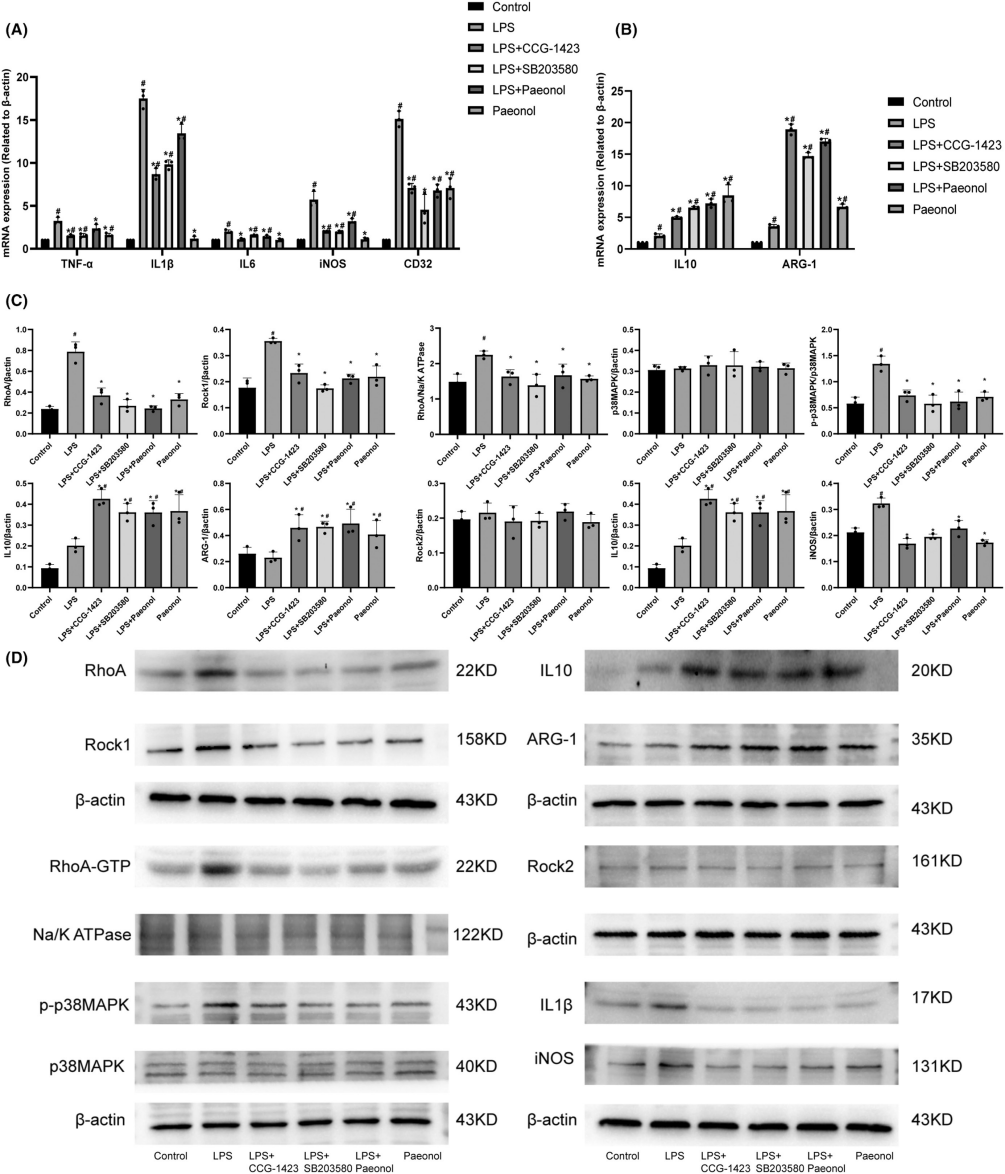
qRT–PCR and Western blot analysis of GMI‐R1 cells. (A) qRT–PCR results of the M1 phenotype proinflammatory factors TNF‐α, IL1β, IL6, iNOS, and CD32. (B) qRT–PCR results of the M2 phenotype anti‐inflammatory factors IL10 and ARG‐1. (C and D) Western blot results of Rhoa/p38MAPK pathway. #Compared with the control group. *Compared with the LPS group. *n* = 3.

To observe the effects of RhoA, p38MAPK inhibitor, and paeonol on inflammatory factors produced by LPS‐induced M1 microglia, we measured the expression levels of IL1β, IL6, TNF‐α, iNOS, CD32, IL10, and ARG‐1. The results showed that the expression levels of proinflammatory cytokines secreted by M1 microglia, such as “IL1β,” “IL6,” and “iNOS,” increased after LPS stimulation, while the cytokines “IL10” and “ARG” secreted by the M2 phenotype were not significantly different from those secreted by the control group. CCG‐1423, SB203580, and paeonol inhibited the expression levels of M1 proinflammatory cytokines (IL1β, IL6, iNOS) (Figure 4A, *p* < 0.05) and increased the expression levels of M2 anti‐inflammatory cytokines (IL10, ARG‐1) (Figure 4B, *p* < 0.05). These results suggest that paeonol can inhibit LPS‐induced secretion of proinflammatory cytokines from GMI‐R1 cells.

### 3.6 Paeonol promotes the transformation of M1 microglia to M2 microglia via the RhoA/p38MAPK signaling pathway

It has been reported that the RhoA/p38MAPK pathway plays an important role in the activation of microglia^20^; therefore, we first observed RhoA, RhoA‐GTP, Rock1, Rock2, p‐p38MAPK, iNOS, IL1β, IL10, and ARG‐1 levels in LPS‐induced GMI‐R1 cells. The levels of RhoA, RhoA‐GTP, Rock1, p‐p38MAPK, iNOS, and IL1β in GMI‐R1 cells were significantly increased after LPS treatment, but there was no significant change in the level of Rock2. CCG‐1423, SB203580, and paeonol decreased the levels of iNOS, IL1β, RhoA, RhoA‐GTP, Rock1, and p‐p38MAPK and increased the levels of Arg‐1 and IL10. The p38MAPK levels were nearly equal between the groups (Figure 3C,D). Paeonol reduced the levels of RhoA‐GTP, Rock1, p‐p38MAPK, iNOS, and IL1β to near normal levels, indicating that paeonol promotes the transformation of M1 microglia to M2 microglia through the RhoA/p38MAPK signaling pathway.

### 3.7 Paeonol alleviates thermal hyperalgesia in CCI model rats

**FIGURE 5:**
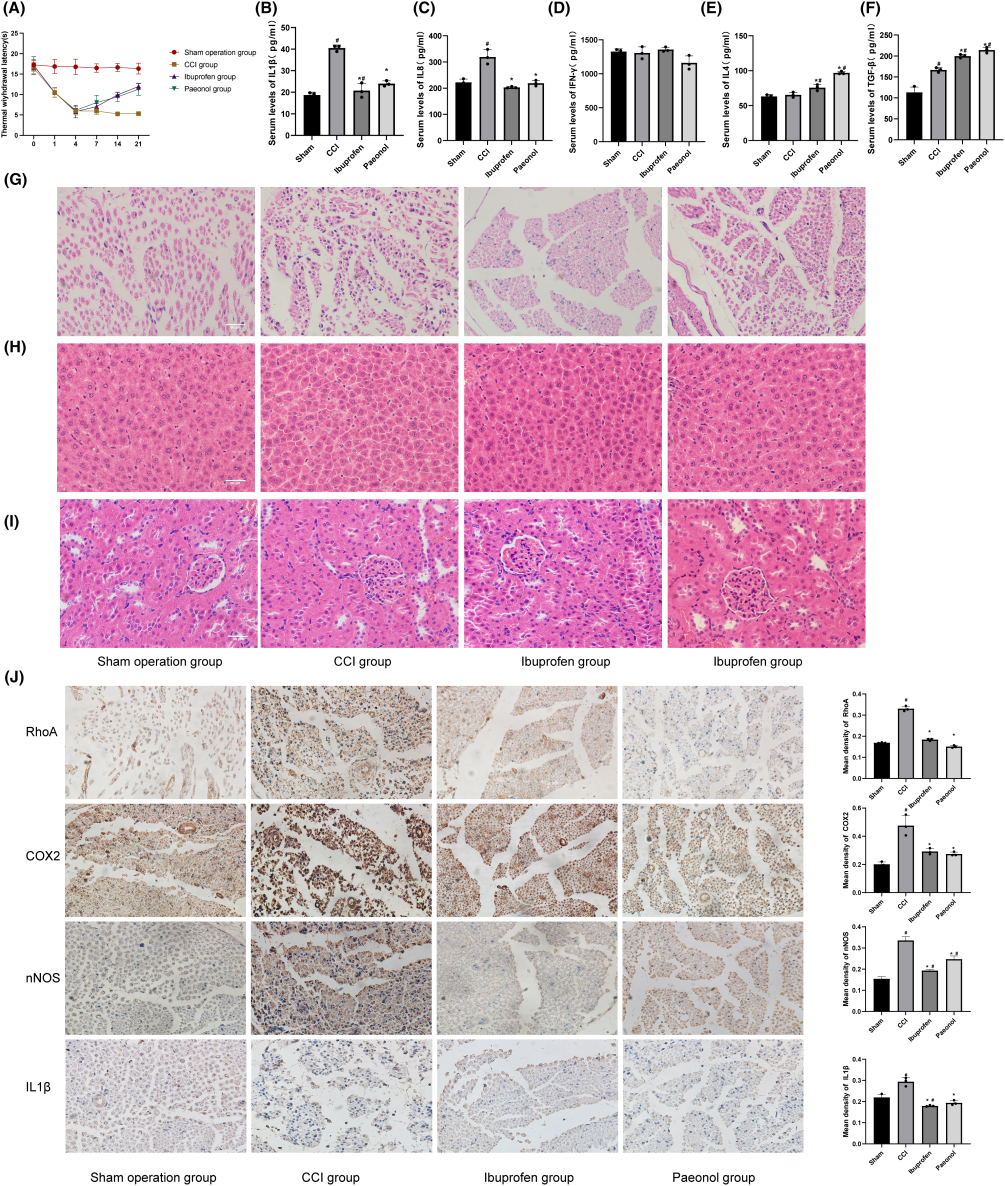
H&E staining, behavioral tests, serum inflammatory factor levels, and sciatic nerve immunohistochemical results. (A) Results of hot plate experiments (*n* = 8). (B–F) Serum inflammatory factor levels (*n* = 3). #Compared with the Sham group. *Compared with the CCI group. *n* = 3. (G) H&E staining of sciatic nerves. Red arrows represent myelin structural destruction, and blue arrows represent inflammatory cell infiltration. (H) Liver H&E staining. (I) Kidney H&E staining. (J) Immunohistochemistry for RhoA, COX2, nNOS, and IL1β. #Compared with the Sham group. *Compared with the CCI group. *n* = 3.

Rats had the most severe thermal hyperalgesia on the 4th day after CCI surgery. The paeonol group and ibuprofen group relieved the thermal hyperalgesia from the 7th day after the treatment but failed to return to normality on day 21. No significant differences were observed between paeonol and ibuprofen (Figure 5A).

### 3.8 Paeonol decreases serum inflammatory factor levels in CCI model rats

Serum concentrations of IL1β, IL8, IFN‐γ, IL4, and TGF‐β were measured. IFN‐γ and IL4 polarized microglia into the M1 and M2 phenotypes. IL1β and IL8 are produced by polarized M1 microglia, while TGF‐β is produced by polarized M2 microglia. The results showed that the serum levels of IL1β, IL8, and TGF‐β in the CCI group were higher than those in the sham group, while the levels of IL4 were not different from those in the sham group. Compared with the CCI group, ibuprofen and paeonol decreased the levels of IL1β and IL8 in serum (Figure 5B,C, *p* < 0.05) and increased the levels of IL4 and TGF‐β (Figure 5E,F, *p* < 0.05). However, there was no difference in IFN‐γ among the four groups (Figure 5D). This finding indicates that paeonol can reduce the level of M1 phenotype proinflammatory factors and increase the level of M2 phenotype anti‐inflammatory factors in the serum of CCI model rats.

### 3.9 Effects of paeonol on the expression of RhoA and inflammatory factors after CCI surgery

We observed sciatic nerve structures by H&E staining. The myelin sheath of the sciatic nerve was destroyed and infiltrated by multiple inflammatory cells after CCI surgery. Myelin structure was restored in the paeonol group and ibuprofen group, and the level of inflammatory cell infiltration was significantly reduced compared with that in the CCI group (Figure 5G). The expression levels of RhoA, COX2, nNOS, and IL1β were observed using immunohistochemistry. COX2 is one of the markers of inflammation and pain, and nNOS is a marker of nervous injury. We found that the expression levels of RhoA, COX2, nNOS, and IL1β increased substantially after CCI surgery. Paeonol and ibuprofen reduced the expression levels of RhoA, COX2, nNOS, and IL1β (Figure 5J). All these findings suggest that paeonol can improve the pathological structure of the sciatic nerve and inhibit the expression of RhoA and inflammatory factors in CCI rats. The liver and kidney structures were normal in all groups (Figure 5H,I), indicating that the administration and CCI operation had no adverse effects on the liver and kidney of rats.

### 3.10 Paeonol reduces the expression of M1 markers and increases the expression of M2 markers in the spinal cord

**FIGURE 6:**
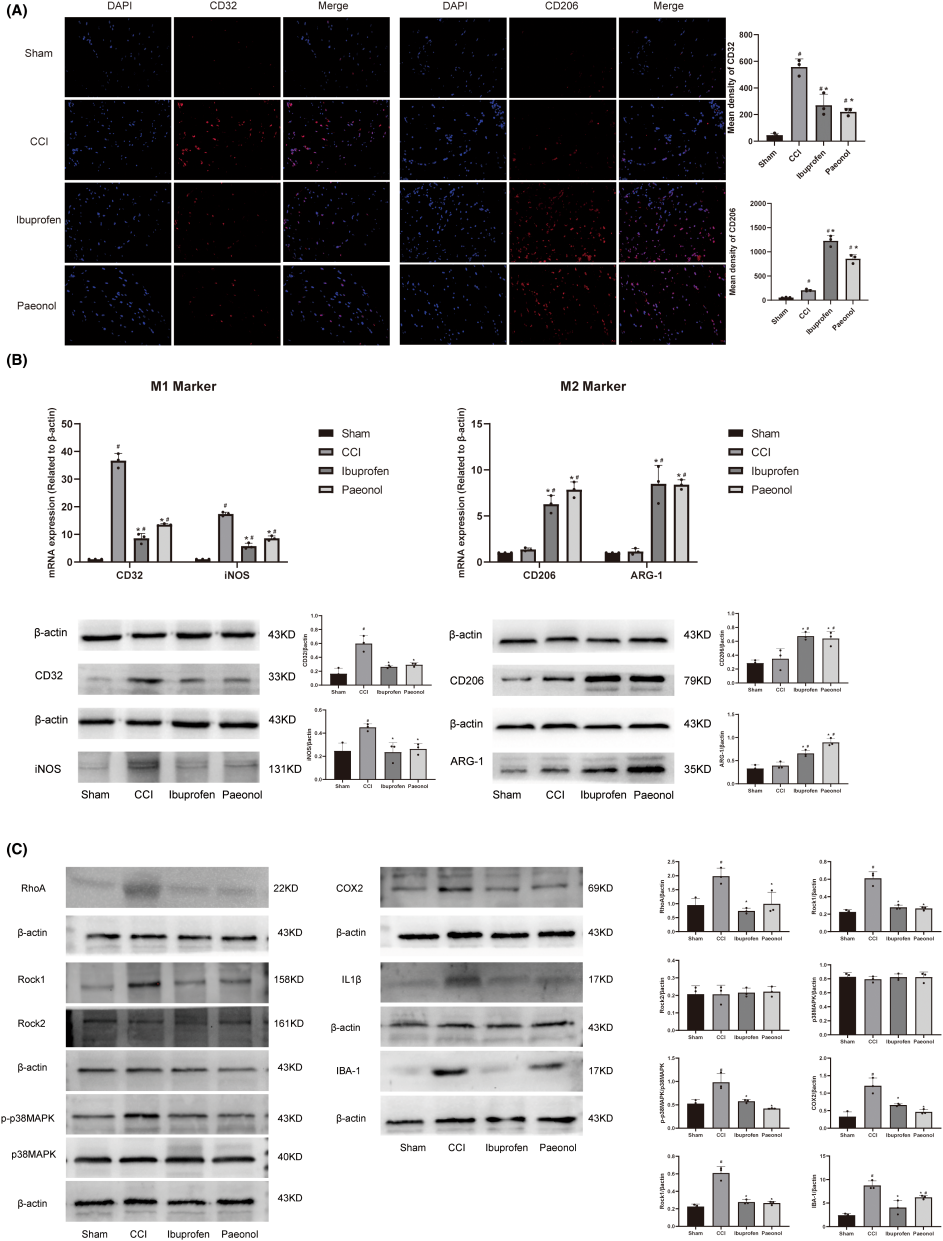
Expression levels of M1 and M2 markers and the RhoA/p38MAPK pathway in the spinal cord. (A) Spinal cord immunofluorescence results for CD32 and CD206. (B) Western blot and qPCR results of expression levels of M1 markers and M2 markers in the spinal cord. (C) Western blot results of RhoA, Rock1, Rock2, p38MAPK, p‐p38MAPK, COX2, IL1β, and IBA‐1 in the dorsal horn of the spinal cord. #Compared to the Sham group. *Compared to CCI Group. *n* = 3.

To further explore whether paeonol can regulate the switch of microglial cells from M1 to M2 phenotype in the spinal cord in vivo, we examined the levels of M1 marker CD32 and M2 marker CD206 in the spinal cord by immunofluorescence. The results showed that the fluorescence intensity of spinal cord CD32 in the CCI model group was higher than that in the sham group, and the fluorescence intensity of spinal cord CD32 in the CCI rats was significantly reduced after ibuprofen and paeonol treatment, but not reduced to the same level as the Sham group. Compared with the sham group, the CD206 fluorescence intensity of the spinal cord of the rats in the CCI model group was slightly increased, while the CD206 fluorescence intensity of the CCI rats was significantly increased after the treatment of ibuprofen and paeonol (Figure 6A). We detected the levels of M1 marker and M2 marker in the spinal cord by qPCR and Western blot. The results showed that compared with the sham group, the expression levels of CD32 and CD206 in the CCI group increased, while paeonol and ibuprofen could reduce the expression levels of CD32 and iNOS. For the M2 markers CD206 and ARG‐1, there was no difference in the expression levels of CD206 and ARG‐1 in the CCI group compared with the Sham group, while ibuprofen and paeonol could significantly increase the expression levels of CD206 and ARG‐1(Figure 6B). This shows that paeonol can switch M1 phenotype microglia to M2 phenotype in the spinal cord of CCI model rats.

### 3.11 Paeonol inhibits the RhoA/p38MAPK pathway and the activation of microglia in the spinal dorsal horn of CCI rats

To understand the expression of the RhoA/p38MAPK pathway in microglia in the spinal cord after sciatic nerve injury and to determine whether microglia are activated, we examined the RhoA/p38MAPK pathway and the microglial activation marker IBA‐1 in the spinal dorsal horn by Western blotting. The results showed that the protein levels of RhoA, Rock1, p‐p38MAPK, COX2, IL1β, and IBA‐1 increased in the CCI group, while paeonol and ibuprofen decreased the expression levels of these proteins. There was no significant difference in p38MAPK and Rock2 among the 4 groups. Paeonol reduced RhoA, Rock1, p‐p38MAPK, COX2, and IL1β levels to near‐normal levels but failed to restore IBA‐1 levels to normal (Figure 6C). This finding indicates that paeonol can reduce neuroinflammation by inhibiting the activation of spinal microglia through the RhoA/p38MAPK pathway.

## 4 DISCUSSION

This study provides evidence that paeonol may relieve sciatica by modulating the polarization of microglia. The main findings are as follows: (1) Differentiation trajectories of spinal cord microglia from peripheral nerve injury revealed that the RhoA/p38MAPK pathway was associated with the polarization of M1 microglia. (2) Paeonol promotes the transformation of M1 microglia to M2 microglia via the RhoA/p38 MAPK signaling pathway. (3) Paeonol reduces peripheral inflammatory factors and reduces neuroinflammation. (4) Paeonol reduces pain caused by neuroinflammation by inhibiting the activation of microglia in the spinal dorsal horn.

Microglia are resident brain immune cells involved in innate immunity.^24^ Growing evidence suggests that microglia play a crucial role in the development and maintenance of neuropathic pain.^25^ Activation of microglia and neuron–glia interactions are considered important mechanisms mediating neuroinflammation and central sensitization.^26^ Central sensitization and neuroinflammation are the main mechanisms leading to persistent hyperalgesia.^27^ When the peripheral nerve is injured, it stimulates the proliferation and M1 polarization of microglia in the dorsal horn of the spinal cord, and M1‐polarized microglia release bioactive substances such as TNF‐α, IL1β, IL6, BDNF, and PGE2.^28^ These inflammatory factors act on spinal dorsal horn neurons and rapidly enhance the strength of excitatory synaptic transmission^29^, ^30^, ^31^ and lead to NMDAR phosphorylation and a reduction in GABA‐mediated synaptic inhibition,^29^ leading to neuronal transition to excitability. In contrast, polarized M2 microglia secrete anti‐inflammatory and neurotrophic factors to repair nerve damage.^32^, ^33^ We found that paeonol can regulate the transformation of M1 microglia to M2 microglia in vitro by flow cytometry and immunofluorescence analysis.

Inflammation is an important factor leading to M1 polarization. LPS and IFN‐γ can induce M1 polarization, and the M1 phenotype secretes proinflammatory factors and aggravates neuroinflammation, forming a vicious cycle.^33^ We found by serum ELISA that paeonol can reduce the serum proinflammatory cytokines IL1β and IL8 and increase the anti‐inflammatory cytokines IL4 and TGF‐β. Through HE staining and immunohistochemical analysis of the sciatic nerve, paeonol was found to reduce the infiltration level of inflammatory cells in the sciatic nerve and the levels of COX2, nNOS, Ilβ, and iNOS in sciatic nerve tissue. These results indicate that paeonol can reduce neuroinflammation and thus relieve pain.

Several studies have shown that the RhoA/p38MAPK pathway promotes the activation of microglia.^18^, ^34^ In this study, microglial trajectory analysis and GMI‐R1 cell experiments confirmed that the RhoA/p38MAPK pathway can lead to the M1 polarization of microglial cells. Studies have shown that Rock activity may be involved in neuropathic, inflammatory, and pain mechanisms.^35^, ^36^, ^37^ Rock modulates neuronal activity by affecting nitric oxide production and plays a key role in microglial activation through p38MAPK.^38^ In this study, we found that the expression of the RhoA/p38MAPK pathway converged with the expression patterns of the M1 activation markers CD32 and CD206 by analyzing the differentiation trajectory of microglia. We found the transformation of LPS‐induced M1 microglia to M2 microglia after adding a RhoA inhibitor (CCG‐1423) and a p38MAPK inhibitor (SB203580) to LPS‐induced M1 microglia. This finding indicates that the RhoA/p38MAPK pathway can promote the M1 polarization of microglia. We found in in vitro experiments that paeonol can reduce LPS‐induced GMI‐R1 microglial RhoA, Rock1 phosphorylated p38, M1 marker CD32, and iNOS protein levels and increase the protein levels of the M2 markers CD206 and ARG‐1. At the same time, we found in in vivo experiments that paeonol can reduce IBA‐1 (microglia activation markers), RhoA, ROCK1, and phosphorylated p38 in the spinal dorsal horn. These results indicate that paeonol can promote the transformation of M1 microglia to M2 microglia through the RhoA/p38MAPK pathway.

Through in vitro and in vivo experiments, we verified that paeonol can reduce neuropathic pain by regulating the M1 and M2 polarization of microglia through the RhoA/p38MAPK pathway, inhibiting central sensitization.

## 5 CONCLUSION

Our study shows that paeonol attenuates central sensitization by modulating the M1 and M2 polarization of microglia, thereby suppressing the resulting neuropathic pain.

## AUTHOR CONTRIBUTIONS

Xin Li, Huimei Shi, Di Zhang, and Guoping Zhao contributed substantially to the experimental design, data analysis, and experimental procedures. Bei Jing, Zhenni Chen, YaChun Zheng, and Shiquian Chang assisted with the English writing. Guoping Zhao is the corresponding author.

## FUNDING INFORMATION

This study was supported by the National Natural Science Foundation of China (grant No. 81874404).

## CONFLICT OF INTEREST STATEMENT

The authors declare no conflicts of interest.

## Data Availability

The data used to support the findings of this study are available from the corresponding author upon request.
